# pH‐Dependent Assembly and Stability of Toll‐Like Receptor 3/dsRNA Signaling Complex: Insights from Constant pH Molecular Dynamics and Metadynamics Simulations

**DOI:** 10.1002/advs.202411445

**Published:** 2024-11-08

**Authors:** Penghui Li, Mingsong Shi, Yibo Wang, Qiong Liu, Xiubo Du, Xiaohui Wang

**Affiliations:** ^1^ Shenzhen Key Laboratory of Marine Biotechnology and Ecology College of Life Sciences & Oceanography Shenzhen University Shenzhen 518055 China; ^2^ Key Laboratory of Optoelectronic Devices and System of Ministry of Education and Guangdong Province College Physics and Optoelectronic Engineering Shenzhen University Shenzhen 518060 China; ^3^ NHC Key Laboratory of Nuclear Technology Medical Transformation Mianyang Central Hospital School of Medicine University of Electronic Science and Technology of China Mianyang Sichuan 621099 China; ^4^ Laboratory of Chemical Biology Changchun Institute of Applied Chemistry Chinese Academy of Sciences Changchun Jilin 130022 China; ^5^ Shenzhen‐Hong Kong Institute of Brain Science Shenzhen Fundamental Research Institutions Shenzhen 518055 China; ^6^ School of Applied Chemistry and Engineering University of Science and Technology of China Hefei 230026 China

**Keywords:** cation‐π Interaction, double strand RNA, molecular dynamics simulations, molecular recognition, Toll‐like receptor 3 (TLR3)

## Abstract

The pH‐dependent assembly of Toll‐like receptors (TLRs), which triggers a threshold‐like response, is a key principle in immune signaling. While crystallography has revealed the intricate structure of these assembly complexes, the mechanisms underlying their pH dependency remain unclear. Herein, constant pH simulations and metadynamics are employed to investigate the pH‐dependent assembly and stability of the TLR3/dsRNA signaling complex. The findings demonstrate that system pH regulates complex assembly and stability by modulating the protonation and charge states of histidines. Histidines in TLR3 act as pH‐dependent, positively charged binding sites that capture negatively charged dsRNA. Additionally, these histidines form a [H682⁺]—[E626⁻] dipole, facilitating the assembly of two TLR3 molecules into an antisymmetric dimer through dipole–dipole interactions. Surprisingly, TLR3 can shift the p*K*_a_ values of key histidines from their model p*K*_a_ of 6.5, increasing protonation likelihood and enhancing ligand binding. Notably, the aromatic residue Phe84, located within the dsRNA binding site [His39⁺–His60⁺–Phe84–His108⁺], alters the p*K*_a_ of His60 through cation‐π interactions with its protonated state. This study offers new insights into the molecular mechanisms underlying pH‐dependent immune signaling via higher‐order assemblies and suggests potential applications for histidine in self‐assembling biomaterials.

## Graphical abstract

Investigate the pH‐dependent assembly of the TLR3/dsRNA signaling complex. Highlights the histidines functions as pH‐dependent, positively charged binding site, and contributing to the dipole that facilitates complex assembly. Propose the protein can modulate the pKa of histidine by selectively interacting with one of its protonation states. Offers a hybrid CpHMD/traditional simulation approach to effectively model biosystems containing hundreds of titratable residues.

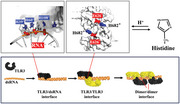

## 1 Introduction

Toll‐like receptors (TLRs) play a pivotal role in innate immunity as transmembrane receptors^[^ ^1^, ^2^, ^3^ ^]^ that detect pathogen‐associated molecular patterns (PAMPs).^[^ ^4^, ^5^, ^6^, ^7^ ^]^ These receptors could recognizing and responding to microbial components by form high‐order assemblies.^[^ ^4^, ^5^, ^6^, ^7^, ^8^, ^9^, ^10^ ^]^ Humans possess ten identified TLR family members, while mice have ≈12.^[^ ^11^, ^12^ ^]^ Each TLR features an extracellular domain for ligand binding, a single transmembrane segment, and an intracellular Toll/IL‐1R (TIR) domain.^[^ ^1^, ^13^ ^]^ The interaction of PAMPs, derived from bacteria or viruses, with the TLR ectodomain can prompt the dimerization of the TIR domains.^[^ ^1^ ^]^ This dimerization is key to initiating downstream signaling pathways that activate immune responses.^[^ ^1^, ^14^ ^]^ TLRs are categorized based on their location and the nature of their ligands: some are situated on the cell membrane, where they respond to lipid or protein ligands, while others, including TLR3, TLR7, TLR8, and TLR9, reside in endosomes and are activated by foreign nucleic acids within these compartments.^[^ ^1^, ^5^, ^6^, ^14^ ^]^

**Figure 1:**
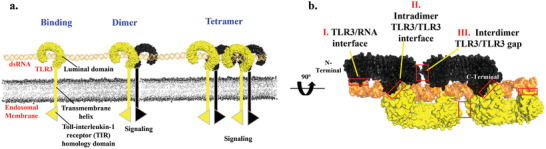
a) The binding of TLR3 onto pathogen dsRNA and the formation of dimer/tetramer signaling complexes. b) The structure of the tetramer signaling complex, formed by the assembly of four TLR3 proteins on double‐stranded RNA (PDB ID: 7WV4). This complex is labeled as TLR3 × 4/dsRNA in our research.

TLR3 plays a critical role in the immune response, primarily located within endolysosomes,^[^ ^15^ ^]^ and activates by recognizing double‐stranded RNAs (dsRNAs) indicative of viral infections or inflammatory processes.^[^ ^7^, ^12^ ^]^ The extracellular domain (ECD) of TLR3 typically exists as a monomer in solution but undergoes assembly on dsRNA to initiate a signaling complex (**Figure** **1**a).^[^ ^16^, ^17^, ^18^ ^]^ This assembly process is crucial for TLR3 activation, depending not on the RNA sequence but rather on the dsRNA's length and the acidic pH conditions (ranging from 5.5 to 6.5 or below).^[^ ^16^ ^]^ For TLR3 to signal effectively, a minimal unit comprising a TLR3 dimer, formed by the cooperative assembly of two TLR3 molecules on a dsRNA segment of 40–50 base pairs, is required.^[^ ^12^, ^19^ ^]^ However, the presence of longer dsRNA strands can promote the formation of tetramers (Figure 1b) or even larger multimeric complexes, enhancing TLR3's activation efficiency.^[^ ^1^, ^7^, ^12^ ^]^ Several efforts have been made to elucidate the mechanism underlying the formation of the signaling complex. Mutations at positions H539E and N541A in TLR3 disrupt its activation and ligand‐binding capabilities, pinpointing these residues as crucial for dsRNA interaction.^[^ ^19^ ^]^ Further structural analyses confirmed their critical location at the TLR3 binding site.^[^ ^7^, ^12^, ^20^ ^]^ Moreover, Coulomb interactions play a crucial role in enabling TLR3 dimers to assemble into higher‐order clusters, essential for effective signaling. This assembly process is facilitated by the attraction between the negatively charged N‐terminal of one TLR3 protein and the positively charged C‐terminal of another, highlighting the importance of these electrostatic forces at the interface of adjacent dimers.^[^ ^12^ ^]^

The pH‐dependent binding of TLR3 to dsRNA highlights a critical aspect of its function, potentially influenced by the pH‐sensitive nature of certain residues. Molecular dynamics simulations offer a method to delve into the atomic‐level behavior of these residues, particularly focusing on their protonation and deprotonation during the ligand‐binding process. However, traditional molecular dynamics simulations, which assume constant protonation states, face significant limitations. These include the requirement for precise knowledge of the p*K*_a_ values^[^ ^22^, ^23^, ^24^ ^]^ of titratable residues to assign correct protonation states accurately. This approach becomes problematic when the p*K*_a_ of a residue is close to the pH of the system, as no single protonation state can fully capture the residue's behavior.^[^ ^25^ ^]^ Furthermore, traditional methods struggle with proton‐coupled dynamics, such as specific protein‐ligand interactions,^[^ ^26^ ^]^ which are crucial in the TLR3/dsRNA signaling complex.

The TLR3/dsRNA complex is particularly challenging to study by using traditional molecular dynamics simulation methods due to its composition of multiple titratable residues — Asp, Glu, His, Lys, Tyr, Cys in the protein, and phosphate and base pair groups in the dsRNA. Notably, ≈ 8 histidine residues per TLR3 molecule are positioned at the interface with dsRNA. Given that the p*K*_a_ of histidine (6.5) is within the active signaling complex's pH range (5.5‐6.5),^[^ ^16^ ^]^ traditional molecular dynamics simulations cannot accurately model histidine's behavior. To overcome these challenges, constant pH molecular dynamics (CpHMD) simulations,^[^ ^26^, ^27^, ^28^, ^29^ ^]^ which dynamically sample protonation states based on the structural context and specified pH, are essential. CpHMD allows for a more accurate investigation of the TLR3/dsRNA complex by accommodating the fluctuating protonation states of residues under different pH conditions.^[^ ^26^ ^]^ This approach is vital for understanding the intricate pH‐dependent interactions within the signaling complex.

**Figure 2:**
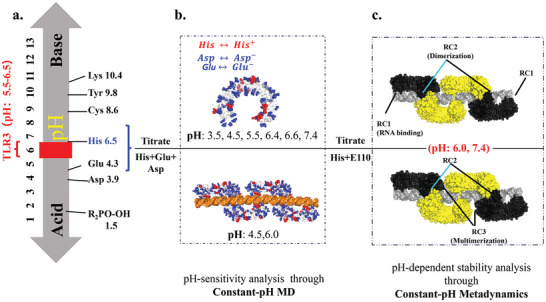
Models constructed in this study are interconnected by threads to reduce the number of residues for titration during CpHMD. a) The study includes a reference^[^ ^21^ ^]^ model p*K*_a_ value for titratable residues and highlights the pH range (in red) pertinent to the active signaling complex formed by TLR3 and RNA. A broader pH range of interest (highlighted in blue) is denoted within the blue bracket. b) Analysis of the pH sensitivity of residues in free TLR3 and the tetramer signaling complex involves titrating all histidine residues (highlighted in red) and aspartate/glutamate residues (highlighted in blue). c) The study employs models to investigate the disassembling of the signaling complex along RC1, RC2, and RC3 (RC: Reaction coordinate; correspond to disassembling along TLR3/RNA interface, intradimer interface, and interdimer interface respectively).

This study began by examining the structural basis of the TLR3 monomer and focus on how it assembles into the TLR3/RNA tetramer^[^ ^12^ ^]^ signaling complex. As shown in **Figure** **2**, we employed a combination of molecular dynamics simulations, constant pH (CpHMD) simulations, and metadynamics. Titration curves for all the Asp, Glu, and His residues in the TLR3 monomer were constructed through a series of constant pH simulations across a range of pH levels (3.5, 4.5, 5.5, 6.5, 6.6, and 7.4). Enabling a detailed examination of how the protonation states and behaviors of these residues vary in different pH conditions. Then, CpHMD simulations were extended to the tetramer signaling complex to explore its stability and dynamics under physiological conditions. Free energy landscapes were calculated for the dissociation of the active signaling complex into monomers at an acidic pH of 6.0. Additionally, we evaluated the effects of a higher pH level, 7.4, on this dissociation process, providing insight into the pH‐dependent dynamics of the TLR3 signaling complex.

## 2 Computational Details

### 2.1 TLR3 Monomer and Tetramer System

The protonation state and p*K*_a_ value of a titratable residue are determined by a combination of its inherent properties and the surrounding environmental conditions. These conditions include the solvent, system pH, and other critical factors such as the proximity of other residues and the presence of ligand molecules. Therefore, to fully understand the pH‐dependent binding of TLR3 to dsRNA, it is essential to analyze both the monomeric form of TLR3 and its structure when bound to a ligand. Furthermore, to gain insights into the interactions between dimers within the signaling complex, this study utilizes the tetramer structure to represent the ligand‐bound state of TLR3, rather than limiting the analysis to the dimer structure.

The structural models for both the TLR3 monomer and the tetramer signaling complex system (donated as TLR3 × 4/dsRNA which formed by four TLR assembling on dsRNA) were derived from the Protein Data Bank (PDB) entry 7WV4.^[^ ^12^ ^]^ This entry provides a detailed representation of a tetramer, where four TLR3 units assemble on a dsRNA molecule with 80 base pairs (bp). Specifically, the TLR3 monomer system was generated by extracting the first TLR3 molecule (chain A) from the complex structure, while the TLR3 × 4/dsRNA tetramer system was created basing on entire structure. Disulfide bonds within the TLR3 protein were managed and constructed using the pdb4amber software, a tool within AmberTools.^[^ ^25^ ^]^

The constructed systems were subsequently solvated in a rectangular box of TIP3P water^[^ ^30^ ^]^ with the minimum distance between the box boundary and solute set to 12 Å. Neutralization was achieved by adding sodium ions. The TLR3 monomer system consisted of ≈145000 atoms, while the tetramer system consisted of ≈440000 atoms. During simulation, the protein atoms were described using the Amber contph force field,^[^ ^28^ ^]^ while the dsRNA was described using the RNA.OL3 force field.^[^ ^31^, ^32^ ^]^ Nonbonded interactions, such as van der Waals and Coulomb interactions, were computed under periodic boundary conditions with a cutoff of 12 Å. The particle mesh Ewald (PME) algorithm^[^ ^33^, ^34^ ^]^ was employed to handle long‐range Coulombic electrostatic interactions.

To prepare both the monomer and tetramer systems for simulation, a sequential relaxation protocol was implemented, consisting of minimization, heating, and further equilibration steps. Initially, the systems were subjected to energy minimization to optimize the water molecules' coordinates. This was achieved by performing 1000 steps of steepest descent optimization, followed by 9000 steps of conjugate gradient optimization, with a restraint applied to all solute atoms. After this initial minimization, the entire systems underwent an additional 10000 steps of optimization to ensure stability. Following the minimization phase, the systems were gradually heated from an initial temperature of 0 K to a target temperature of 300 K over a period of 25 picoseconds (ps), employing the NVT ensemble to maintain constant volume and temperature. The final phase of the relaxation process involved equilibrating the systems under the isothermal–isobaric (NPT) ensemble for 750 ps, ensuring the pressure remained constant at 1 atm. This comprehensive relaxation protocol was designed to stabilize the systems and accurately reflect physiological conditions.

### 2.2 Constant pH MD Simulations

After setup, both the monomer and tetramer systems were then prepared for constant pH simulations. Constant pH molecular dynamics (CpHMD) simulations were conducted to investigate the pH sensitivity of titratable residues and the mechanism underlying the pH‐dependent assembly^[^ ^16^ ^]^ of the signaling complex within the pH range of 5.5 to 6.5. During simulation, all Asp, Glu, and His residues were titrated with flexible protonation states, since their p*K*_a_ values are close to the pH range of 5.5 to 6.5 (Figure 2a, p*K*_a_ values of Asp, Glu, and His in alanine pentapeptides: 3.9, 4.3, 6.5).^[^ ^21^ ^]^ However, other titratable residues such as Lys, Tyr, and Cys in the protein, as well as the R_2_PO─O^─^ group in dsRNA, were assigned a single protonation state, as their p*K*_a_ values are far from the pH range of 5.5 to 6.5 (p*K*_a_ values of Lys, Tyr, and Cys in alanine pentapeptides: 10.4, 9.8, 8.6).^[^ ^21^ ^]^

Multiple CpHMD simulations with different pH values were conducted to investigate the pH sensitivity of titratable residues in both the TLR3 monomer and the signaling complex (Figure 2b). This included six simulations of the TLR3 monomer with pH values set to 3.5, 4.5, 5.5, 6.4, 6.6, and 7.4, respectively, and one simulation of the TLR3 × 4/dsRNA signaling complex with pH set to 4.5. During CpHMD, the protonation states of titratable residues were attempted to be reassigned every 500 MD steps, followed by a relaxation period of 200 MD steps before continuing the simulation. For the CpHMD simulations, the TLR3 monomer systems were simulated for a duration of 1000 ns each. Meanwhile, the TLR3×4/dsRNA signaling complex at pH 4.5 was subjected to a CpHMD simulation time of 160 ns.

### 2.3 Metadynamics Simulations

To unravel the intricate mechanism behind the pH‐dependent assembly of the TLR3 × 4/dsRNA signaling complex, it's crucial to explore the free energy landscape along its assembly or disassembly pathway. The presence of free energy barriers between different configurational states necessitates the use of enhanced sampling techniques to make the computational exploration feasible and cost‐effective. Various enhanced sampling methods have been developed to study biological processes' free energy landscapes, including umbrella sampling,^[^ ^35^ ^]^ metadynamics,^[^ ^36^, ^37^ ^]^ thermodynamic integration,^[^ ^38^, ^39^, ^40^, ^41^, ^42^, ^43^ ^]^ targeted MD,^[^ ^44^ ^]^ and steered MD.^[^ ^45^ ^]^ In this study, we utilized the well‐tempered metadynamics approach, a refined version of metadynamics that ensures asymptotic convergence by applying a history‐dependent Gaussian potential to guide the system's evolution in phase space.^[^ ^36^, ^37^ ^]^ This method was strategically integrated with constant pH simulations to enable a thorough analysis of the signaling complex's assembly dynamics under varying pH conditions.

The selection of well‐chosen collective variables (CV) acting as reaction coordinates (RC) is crucial for describing the assembly or disassembly states of the TLR3 × 4/dsRNA signaling complex, as well as for subsequent metadynamics simulations aimed at exploring the free energy landscape. As shown in Figures 1b and 2c, the assembly‐disassembly dynamics of the TLR3 × 4/dsRNA signaling complex are governed by interactions across three key interfaces: intradimer interface between TLR3 proteins within a single dimer, interdimer interface between adjacent dimers, and dsRNA/TLR3 interface between TLR3 and dsRNA. To accurately describe these processes, we can utilize coordination numbers (CN) as collective variables (CVs). These variables are effective in capturing the essence of the interactions among different molecular groups. Specifically, the coordination numbers are defined and calculated within the plumed software as follows:

(1) $$CN_{ij}=\frac{1-{\left(\frac{r_{ij}-d_{0}}{r_{0}}\right)}^{6}}{1-{\left(\frac{r_{ij}-d_{0}}{r_{0}}\right)}^{12}}\;$$

(2) $$CN_{Total}=\sum_{i\in A}\sum_{j\in B}CN_{ij}\;$$

where *CN_ij_* is the coordination number between the *ith* atom in group A and the *jth* atom in group B, *r_ij_* is the distance (unit in Å) between the *ith* atom in group A and the *jth* atom in group B,.*CN_Total_* is the total coordination number between atoms in group A and atoms in group B, *d*_0_ =  0, and the *r*_0_ parameter of the switching function which set as 3.0 Å in this work.

Three sets of coordination numbers chosen as reaction coordinate (RC) for the meta‐dynamics simulations for TLR3×4/dsRNA signaling complex. The first reaction coordinate (RC1) is the coordination number between the four TLR3 and the dsRNA, denote as *CN*_(*TLR*3 − *RNA*)_, which describes the binding/dissociation of TLR3 onto dsRNA. The second reaction coordinate (RC2) is the total intradimer coordination number between different TLR3 in one dimer, denote as *CN*_(*TLR*3 − *TLR*3)_, which describes the assemble/disassemble of TLR3 in dimer. The third reaction coordinate (RC3) is the interdimer coordination number between nearby dimers denote as *CN*_(*dimer* − *dimer*)_, which represents the interaction that drive the assembly of dimer into tetramer.

To explore the pH‐dependent assembly dynamics of the TLR3×4/dsRNA signaling complex, four CpHMD metadynamics simulations were conducted. The simulations were structured as follows: i) Metadynamics simulation along RC1 and RC2 with system at pH 6.0, ii) Metadynamics simulation along RC1 and RC2 with system at pH 7.4, iii) Metadynamics simulation along RC2 and RC3 with system at pH 6.0, and iv) Metadynamics simulation along RC2 and RC3 with system at pH 7.4. To optimize computational efficiency, the simulations included only 92 titratable residues, comprising all histidine (His) residues within the TLR3 protein and the E110 residue located at the TLR3‐dsRNA interface. Throughout these simulations, Gaussian hills with a height of 1.2 kJ mol^−1^ were added at intervals of 0.2 ps to modulate the free energy landscape. The Gaussian hills were applied with widths of 25 units for both dimer‐dimer (*CN*_(*dimer* − *dimer*)_) and TLR3‐TLR3(*CN*_(*TLR*3 − *TLR*3)_) interactions, and a width of 50 units for TLR3‐dsRNA (*CN*_(*TLR*3 − *dsRNA*)_) interactions. The calculations were carried out in a well‐tempered ensemble^[^ ^36^, ^37^ ^]^ with the bias factor set as 25. To ensure convergence, the simulations were performed for a total of 250, 282, 220, and 150 ns for (i), (ii), (iii), and (iv) system respectively. The duration of metadynamics simulations varies due to the unique potential of mean force (PMF) landscapes associated with each system. Since metadynamics fills PMF wells with Gaussian potentials, shallower wells result in shorter convergence times. The time series curves for the PMFs are shown in Figure S1 (Supporting Information) (ESI†), indicating that the phase spaces related to the assembly process were effectively sampled. Notably, the PMF exhibits minimal changes during the final phase of the simulation. Further convergence analyses were conducted using the block‐analysis technique implemented in PLUMED software, which provides an ensemble average method to estimate computational error. Convergence was confirmed upon the observation of plateaus in the respective plots (Figure S1, Supporting Information).

All constant pH simulations and constant pH Metadynamics simulations were performed under NVT ensemble with the temperature set as 300 K by using a Langevin dynamics thermostat.^[^ ^46^ ^]^ The bond‐stretching freedom which involves hydrogen atoms were constrained by the SHAKE algorithm.^[^ ^47^ ^]^ The Velocity–Verlet algorithm^[^ ^48^ ^]^ was adopted for the MD simulations with 2 fs time step. All the MD simulations were performed using the GPU version (pmemd.cuda) Amber22 suite,^[^ ^25^ ^]^ and the Metadynamics simulations were performed by combine Amber22 and plumed‐2.8.0.^[^ ^49^, ^50^, ^51^ ^]^ The trajectories were visualized using Open‐Source PyMOL.^[^ ^52^ ^]^

## 3 Results and Discussion

### 3.1 pH Sensitivity Analysis and p*K*_a_ Prediction

pH sensitivity analysis^[^ ^53^, ^54^, ^55^ ^]^ is essential for assigning the correct protonation states of titratable residues during molecular simulations. The accuracy of these protonation states critically impacts Coulomb interactions during the assembly of the charged TLR3 onto negatively charged dsRNA. However, accurate pH sensitivity analysis is challenging because the p*K*_a_ of ionizable residues is influenced by their surrounding environment and can evolve during the ligand‐binding process.^[^ ^27^ ^]^ To address this, we conducted four sets of pH sensitivity analyses to compare the accuracy of two major methods and examine p*K*_a_ differences between the monomer and tetramer states. The analysis includes one cost‐effective PROPKA^[^ ^24^, ^56^, ^57^, ^58^, ^59^ ^]^ method and three computationally expensive explicit‐solvent CpHMD^[^ ^53^, ^60^, ^61^ ^]^ simulations. The three CpHMD approaches consist of one CpHMD titration method, involving six CpHMD simulations, and two single CpHMD simulations that predict p*K*_a_ based on the protonation ratio of each residue, with system pH set to 4.5 and 6.0.

#### 3.1.1 Monomer

p*K*_a_. As shown in **Table** **1**, both the PROPKA and CpHMD methods predict p*K*_a_ values for most residues that closely match their model p*K*_a_ values(p*K*_a, His_ = 6.5, p*K*_a, Asp_ = 3.9, and p*K*_a, Glu_ = 4.3,^[^ ^21^ ^]^ Δ p*K*_a_ <1). This suggests that the PROPKA method can provide reasonable p*K*_a_ values similar to the CpHMD titration method for residues with non‐unique surroundings. However, notable p*K*_a_ shifts (Δ p*K*_a_ >1) are observed between PROPKA and CpHMD for some residues. For example, E211 (p*K*_a_: 4.5→5.6), E239(p*K*_a_: 4.5→5.6) and H108 (p*K*_a_: 5.7→6.9). Additionally, the CpHMD titration method (Figure S2, Supporting Information) demonstrates that the titration curves for some residues do not fit a sigmoid model. This indicates a strong coupling effect with nearby polar or charged residues. Although PROPKA shown p*K*_a_ prediction issues across His, Asp, and Glu, the errors do not significantly affect the dominant protonation state for most Asp/Glu, except His, residue in mild pH environments (pH 6–8).

**Table 1:** Predicted p*K*_a_ values for residues in TLR3 using four methods/states.

His					Asp/Glu							
	Monomer		Tetramer			Monomer		Tetr		Monomer		Tetr
	PR^a)^	Titr^b)^	pH_4.5_ ^c)^	pH_6.0_ ^d)^		PR	Titr	pH_4.5_		PR	Titr	pH_4.5_
H32	6.4	6.2	5.7	6.3	E33	4.5	4.5	4.7	D366	3.8	<3.5	3.8
H39	6.2	6.9	8.3	8.0	D36	3.8	<3.5	3.4	E399	4.5	4.7	4.9
H60	5.6	o^e)^	‐^f)^	8.2	D47	3.8	<3.5	3.8	E423	4.5	<3.5	4.4
H108	5.7	6.9	8.5	8.1	D48	3.8	4.5	4.5	D425	3.8	<3.5	3.2
H129	8.1	no	4.2	6.1	D81	3.8	<3.5	3.6	E434	4.5	4.9	4.7
H156	5.9	5.2	6.9	7.1	E91	4.5	3.5	4.4	D437	3.8	no	5.0
H218	6.6	7.4	7.2	9.4	E93	4.5	no	4.6	E442	4.5	4.0	4.7
H312	6.8	4.5	5.0	5.8	E110	4.5	5.0	5.7	E446	4.5	4.0	4.5
H316	6.5	no	6.0	5.9	D116	3.8	<3.5	3.8	E451	4.5	<3.5	3.2
H319	5.8	6.8	5.7	7.3	E127	4.5	no	4.6	E456	4.5	5.1	5.1
H359	5.2	4.8	6.0	6.6	D153	3.8	<3.5	1.6	E460	4.5	3.7	4.4
H406	6.4	6.3	6.0	6.5	E171	4.5	3.9	4.3	D496	3.8	<3.5	4.3
H410	5.9	5.8	5.1	6.5	E175	4.5	4.8	5.0	D512	3.8	<3.5	2.2
H432	6.3	6.5	5.5	6.3	E189	4.5	<3.5	3.9	D523	3.8	no	3.2
H539	5.9	no	8.7	8.3	E190	4.5	5.0	5.2	D524	3.8	5.3	4.8
H548	6.6	6.1	6.0	7.8	D192	3.8	no	4.1	E527	4.5	no	4.4
H563	6.5	No	6.1	7.8	E203	4.5	<3.5	3.7	E530	4.5	4.3	4.4
H565	6.9	5.9	5.0	6.7	E211	4.5	5.6	4.5	E533	4.5	no	4.1
H665	6.4	6.5	6.1	6.6	E239	4.5	5.6	4.4	D536	3.8	<3.5	3.5
H674	6.1	6.8	7.1	8.9	E244	4.5	no	3.4	E570	4.5	<3.5	4.7
H682	6.3	6.5	5.9	7.1	D280	3.8	<3.5	2.5	D575	3.8	<3.5	4.2
H684	6.3	6.5	6.6	7.1	D292	3.8	<3.5	3.6	E576	4.5	no	4.5
					E301	4.5	4.5	4.3	E580	4.5	4.5	4.6
					E306	4.5	4.0	4.7	D584	3.8	no	4.4
	PRO	Titr	pH_4.5_		D347	3.8	<3.5	3.5	E587	4.5	<3.5	4.3
D648	3.8	<3.5	2.3		D348	3.8	<3.5	3.3	D592	3.8	5.0	4.4
E652	4.5	5.2	4.8		E358	4.5	<3.5	3.4	E626	4.5	no	4.8
E663	4.5	5.3	5.3		E363	4.5	<3.5	3.3	E639	4.5	no	5.0
E670	4.5	No	5.1		D364	3.8	<3.5	3.8	D641	3.8	<3.5	2.5

^a)^ PROPKA: Predicted by PROPKA software for the TLR3 monomer (Chain A of the tetramer signaling complex, PDB id: 7wv4);

^b)^ Titration Curve: Predicted by simulated titration curves shown in Figure S1 (Supporting Information). Six constant pH molecular dynamics simulations were conducted with system pH values set at 3.5, 4.5, 5.5, 6.4, 6.6, and 7.4. Each CpHMD simulation was performed for 1 µs;

^c)^ Tetramer p*K*_a_: Averaged value for the four residues in the four TLR3 subunits. Predicted by a single CpHMD simulation of 160 ns with the system pH set at 4.5;

^d)^ Tetramer p*K*_a_: Predicted by a single 160 ns CpHMD simulation before metadynamics with the system pH set at 6.0. Only His and E110 were titrated in this system. The p*K*_a_ for E110 is 6.6;

^e)^ No p*K*_a_ value is provided for these residues as their titration curves do not fit a sigmoid model;

^f)^ No p*K*_a_ value is provided for these residues as they exhibited only one protonation state during the CpHMD simulation.

#### 3.1.2 p*K*_a_ Shift in Tetramer

The estimated p*K*_a_ values for the 84 titratable His, Glu, and Asp residues in the tetramer signaling complex, as determined by single CpHMD simulations at pH 4.5 and 6.0, are shown in Table 1. The p*K*_a_ predictions from CpHMD at pH 4.5 are similar to those at pH 6.0. Meanwhile, significant p*K*_a_ shifts are observed when comparing the TLR3 tetramer to the TLR3 monomer, particularly among histidine residues at the assembling interface (see Figure S3, Supporting Information). The TLR3/dsRNA binding interface contains histidines H39, H60, H108, and H539, the dimerization interface includes H674, H682, and H684, while the tetramerization interface between two dimers does not contain any histidines. The p*K*_a_ values for H39, H60, H108, and H539 at the dsRNA binding interface of TLR3 increase from ≈6.0 to 8.0/8.3, ∞/8.2, 8.5/8.1, and 8.7/8.3, respectively, as determined from the cpHMD tetramer system at pH 4.5/6.0. Additionally, the p*K*_a_ value for H674 at the dimerization interface also rises to 7.1/8.9. The typical Δp*K*_a_ ≈ +2 for those histidines suggests that the protonation ratio of these histidine residues increases by ≈ 100‐fold when binding to negatively charged dsRNA, since the Coulomb interaction at the interface formed by charged or protonated histidines discourages the non‐protonated state.

Although the estimated p*K*_a_ values for several Asp and Glu residues also show significant shifts (Δp*K*_a_ > 1), most predicted values remain below 5.0. This suggests that within the pH range of 5.5 to 6.5, these residues are likely to remain deprotonated. Consequently, these shifts have minimal impact, as they do not affect the assignment of the protonation state. In contrast, the p*K*_a_ values for E110 in the tetramer are 5.7/6.6. This variability indicates that E110 cannot be assigned a single protonation state throughout the ligand‐binding simulation. Its distinct protonation dynamics could significantly influence its interaction with dsRNA, underscoring the importance of considering individual residue behaviors in complex formations.

#### 3.1.3 p*K*_a_ Shift for Histidines

**Figure 3:**
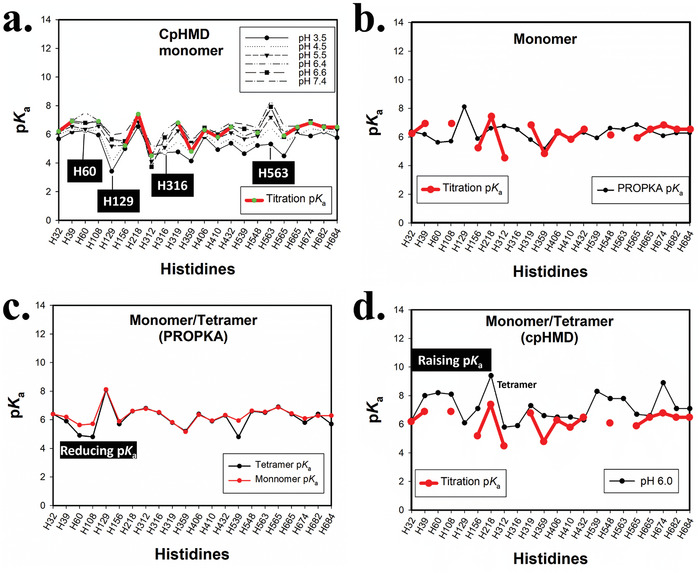
The shifts between different estimated p*K*_a_ values for histidines across monomer and tetramer states, as well as between the PROPKA and CpHMD methods: a) The p*K*_a_ values for histidines estimated using the titration CpHMD method across six simulations (red), compared with the apparent p*K*_a_ values result from the protonation ratio of each individual CpHMD simulation (black). Histidines with non‐standard titration curves, as shown in Figure S2 (Supporting Information), are highlighted with black boxes. b) The shifts in estimated p*K*_a_ values between the PROPKA method and the titration CpHMD method. c) The shifts in estimated p*K*_a_ values between the monomer and tetramer states as predicted by the PROPKA method. d) The shifts in estimated p*K*_a_ values between the monomer (red) and tetramer (black) states as predicted by the titration method and the 160 ns of CpHMD simulation before metadynsmics. The statistical analysis for p*K*_a_ values calculation form protonation ratio was performed using cphstats script in AmberTools.

As mentioned above, the estimated p*K*_a_ values for several histidines suffers intense shift across monomer/tetramer state and PROPKA/CpHMD method. The titration curve for H60, H129, H316, and H563 cannot fit into standard sigmoid titration curve (Figure S1, Supporting Information), and the apparent p*K*_a_ values for those histidines were disturbed (**Figure** **3**a). And the PROPKA result show significant p*K*_a_ shifts (Δ p*K*_a_ >1) for those histidines. As for shifts between the p*K*_a_ for monomer and tetramer, although both PROPKA and CpHMD method predict there is significant shift for some histidines, but the predicted shift tend is contrary (Figure 3c,d), Which imply the PROPKA method could cause problem in some case. Thus, CpHMD method rather than PROPKA method should be adopted during the simulation for pH‐dependent process when possible.

#### 3.1.4 Hybrid CpHMD/Traditional Simulation

Based on the pH sensitivity analysis discussed above, a hybrid CpHMD/traditional simulation method was employed, where some residues were assigned a constant protonation state while others were handled by the CpHMD method. This approach was used for the subsequent metadynamics simulations in the next section. All histidines were treated with the CpHMD method, as their p*K*_a_ values and apparent p*K*_a_ values are near the pH range of interest (5.5–6.5). All Asp and Glu residues, except for E110, were assigned a non‐protonated state due to their p*K*_a_ values being less than 5.0 in both the monomer and tetramer states. E110 was also handled by the CpHMD method, as its p*K*_a_ value can increase to 5–6 when binding to dsRNA in the tetramer, a result of the Coulombic repulsion between E110 and dsRNA.

### 3.2 pH‐Dependent Stability Analysis by Metadynamics

Based on the hybrid CpHMD/traditional system constructed after the pH sensitivity analysis, this study investigated the pH‐dependent stability of the signaling complex using metadynamics simulations. The disassembly of the signaling complex was examined along three reaction coordinates (RCs) under two different pH environments (6.0 and 7.4). The pH value 6.0, falling at the midpoint of the pH range (5.5 to 6.5), is the typical value for an active signaling complex; while pH 7.4, which would result in the disassembling of signaling complex, representing a classical basic environment in living organisms. As mentioned above, RC1 and RC2 drive the disassembling of the signaling complex along TLR3/dsRNA and intradimer TLR3/TLR3 interface respectively, while RC3 drive the disassembling along interdimer TLR/TLR3 interface.

#### 3.2.1 Stable at pH 6.0 (RC1 and RC2)

**Figure 4:**
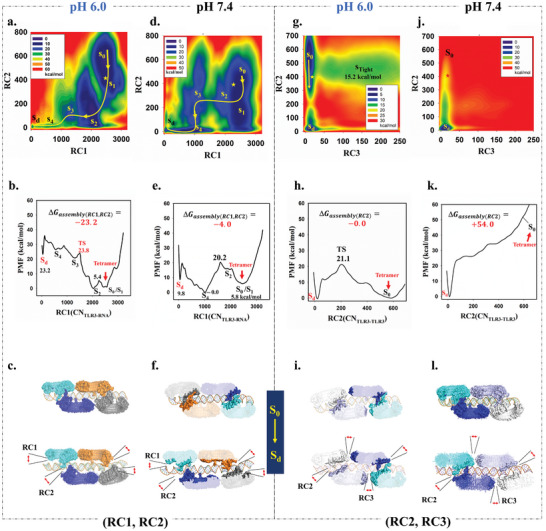
The potential of mean force (PMF, upper), 1D minimum free energy path (MEP, middle), and some typical snapshots from metadynamics (bottom) depict the disassembly of the signaling complex along reaction coordinates (RC1/RC2, RC2/RC3) at pH 6.0 or pH 7.4. The location of reaction coordinate for initial structure (PDB ID: 7WV4) is marked with a yellow or red star on the PMF plots. S_0_ denotes the first local minima where crystal tetramer is located, S_d_ denotes the disassembled state. The states S_1_, S_2_, S_3_, and S_4_ refer to intermediate states. a–c) Along RC1/RC2 at pH 6.0. d–f) Along RC1/RC2 at pH 7.4. g–i) Along RC2/RC3 at pH 6.0. j–l) Along RC2/RC3 at pH 7.4. The statistical analysis for PMF was performed using the sum_hills module in PLUMED with the command: plumed sum_hills –hills HILLS –mintozero –bin 99,99.

The potential of mean force (PMF) for disassembling of the TLR3 signaling complex along RC1 and RC2 at pH 6.0 is illustrated in **Figure** **4**a. The minimum energy path (MEP), shown in Figure 4b, revealed five local minima transitioning from the fully assembled signaling complex to a disassembled monomer state. This disassembly pathway, represented as *S*_0_↔*S*_1_↔*S*_2_↔*S*_3_↔*S_d_* (Figure 4c), indicated the sequential breakdown of the complex. The overall free energy change associated with the complete disassembly is 23.2 kcal mol^−1^. This complex disassembly can be conceptually divided into two main steps, differentiated by their major projection along RC1 or RC2 on the MEP. The initial step, denoted as *S*_0_↔*S*_1_↔*S*_2_, progressed primarily along RC2, focusing on the separation of TLR3 from its adjacent counterparts, with few free energies change and encountering an energy barrier of 5.4 kcal mol^−1^. The subsequent step, denoted as *S*_2_↔*S*_3_↔*S_d_*, primarily drove the disassembly of TLR3 with dsRNA, requiring a significantly higher free energy change of ≈23.2 kcal mol^−1^ and overcoming an energy barrier of 23.8 kcal mol^−1^. These results highlight that separating TLR3 from dsRNA poses a greater challenge than dissociating TLR3 units within the signaling complex. This implies that at pH 6.0, the TLR3 signaling complex is more likely to spontaneously assemble, particularly through the interaction of TLR3 with dsRNA.

#### 3.2.2 Unstable at pH 7.4 (RC1 and RC2)

The reason why signaling complex needs an acid environment is a key issue. The potential of mean force (PMF) for the disassembly of the signaling complex along RC1 and RC2 at pH 7.4 is depicted in Figure 4d. The shape of PMF exhibits some similarities with the PMF in Figure 4a, as the only difference between the two systems is the pH value. Along the minimum energy path (Figure 4e), ≈ 6 major local minima can be observed from the assembled signaling complex to the disassembled monomer, denoted as *S*_0_↔*S*_1_↔*S*_2_↔*S*_3_↔*S*_4_↔*S_d_*, representing the disassembly process of the signaling complex (Figure 4f).

However, the rise of system pH from 6.0 to 7.4 results in a significant and abrupt change in the relative depth of the local minima. The total free energy difference from assembly to disassembly changes from +23.2 kcal mol^−1^ (at pH 6.0) to +4.0 kcal mol^−1^ (Figure 4e), indicating the disappearance of strong interactions that drive the formation of the signaling complex in base environment. Moreover, the overall minima are no longer situated in the S_1_ assembled signaling complex state with (RC1, RC2) coordinates ≈ (2500, 400) but situate in the partly disassembled S_4_ state with (RC1, RC2) coordinates ≈ (1000, 0). The RC1 value in the S_4_ state decreases by 60% from 2500 to 1000, indicating that the binding of TLR3 onto dsRNA is much weaker at pH 7.4 than at pH 6.0. The RC2 value in the S_4_ state is around zero, indicating that TLR3 would be unable to assemble into dimers at pH 7.4.

#### 3.2.3 Stable at pH 6.0 (RC2 and RC3)

The potential of mean force (PMF) depicting the disassembly of the tetrameric signaling complex along RC2 and RC3 at pH 6.0 is illustrated in Figure 4g. Three local minima are discernible in this PMF, which represent distinct states along the disassembly pathway. The *S_Tight_* ↔*S*_0_↔*S_d_* process could be viewed as the route for the system to transition from the tightly assembled signaling complex S_Tight_ to the disassembled state S_d_.

The assembly of the system along RC2 and RC3 should be viewed as two independent processes, because the *S*_1_↔*S*_0_ route is mainly evolving along RC3 thus orthogonal with the *S*_0_↔*S_d_* route which mainly evolves along RC2. This suggests that the assembly of two TLR3 proteins into dimers is not directly synergic with the assembly of two dimers into tetramers route along RC2. Thus, the minimum energy path (MEP) along RC2 represents the *S_d_* ↔*S*_0_ rate‐limiting step (Figure 4h), which indicates that the assembly of TLR3 on dsRNA into a dimer is a slightly exothermic reaction (1.2 kcal mol^−1^) with an energy barrier of 21.1 kcal mol^−1^. However, the minimum energy path (MEP) along RC3 (Figure S4a, Supporting Information), which governs the movement of the interdimer interface, undergoes a transformation from +15 kcal mol^−1^ (RC3 = 0) to 0 (RC3 = 10) kcal mol^−1^ to +15.2 kcal mol^−1^ (RC3 = 150). This illustrates that the assembly and draw near of the dimer into a tetramer halt at RC3 = 10, where only 10 pairs of atoms in two dimers stay within 3 Å distance. Conversely, distances that are too close or too far between the two dimers result in an ≈15.2 kcal mol^−1^ increase in the system's free energy. The structure of S_0_ and S_d_ states were shown in Figure 4i.

#### 3.2.4 Unstable at pH 7.4 (RC2 and RC3)

The potential of mean force (PMF) for the disassembly of the tetramer signaling complex along RC2 and RC3 at pH 7.4 is depicted in Figure 4j. The number of local minima decreased from three (at pH 6.0) to one, and the overall minimum representing the disassembled state (S_d_) at coordinates (RC2/RC3 = 10/0). The minimum energy path (MEP) along RC3, shown in Figure S4b (Supporting Information), suggests that the assembly of the TLR3 dimer interface might not be strongly affected by pH, since the energy for the disassembled state along RC3 is also ≈ 15 kcal mol^−1^. However, the energy for the tightly assembled state rises to +50 kcal mol^−1^ (Figure S4b (Supporting Information), RC3 = 200). On the other hand, the assembly of TLR3 into a dimer along RC2 is severely disrupted. As depicted in Figure 4k, the free energy for the assembled state (S_0_) increases to +54.0 kcal mol^−1^, indicating that the dimer or intradimer interface cannot exist at pH 7.4.

### 3.3 Mechanism for pH‐Dependent Stability: Charged Histidines form Fingerprint for Assembling

As discussed above, this study successfully reproduced the pH‐dependent stability of the TLR3×4/dsRNA signaling complex observed in experiments. This validates the model system we constructed and demonstrates that the hybrid CpHMD/traditional method can accurately describe the real signaling complex. We can now proceed to investigate the atomic mechanisms underlying the pH‐dependent stability.

#### 3.3.1 Charged Histidines

In the ideal model, ≈76% of histidine residues are protonated and charged at pH 6.0, while ≈ 11% are protonated and charged at pH 7.4, based on the following Equations (3) and (4):

(3) $$His+H^{+}↔\;His^{+}$$

(4) $$k_{a}=\frac{{C_{His}}^{+}}{{C_{H}}^{+}\times C_{His}}\approx 10^{6.5}$$

This suggests that an acidic environment could enhance the His^+^ state of histidine residues, thereby promoting the stability of the signaling complex. However, the overall charge state of TLR3 alone does not account for the complex's orderliness. Therefore, a detailed understanding of the role and protonation state of histidines in the signaling complex is necessary. As mentioned previously, the constant pH molecular dynamics (CpHMD) approach was employed during the metadynamics simulation to manage the protonation states of 88 histidine residues and four E110 residues. In total, 92 residues were assigned flexible protonation states within the signaling complex.

**Figure 5:**
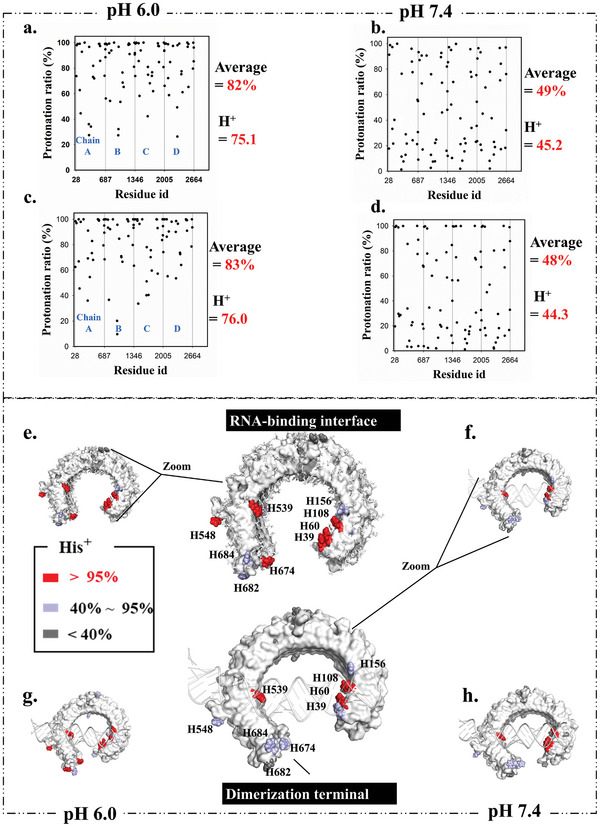
Protonation ratio for all the histidine residues during the constant pH (6.0, 7.4) metadynamics simulations for the disassembling of the signaling complex along routes RC1/RC2 and RC2/RC3. a–d) Protonation ratio of all titrated residues during the disassembling process along RC1/RC2 (left) and RC2/RC3 (right). e–h) Protonation ratio of histidine residues during the disassembling process along RC1/RC2 (left) and RC2/RC3 (right) which is colored by residue protonation ratio. The statistical analysis for protonation ratio was performed using cphstats script from AmberTools.

As illustrated in **Figure** **5**a–d, ≈(82%, 49%, 83%, 48%) of the 92 titrated residues were protonated during the four CpHMD metadynamics simulation respectively. The overall protonation ration strongly relates with system pH but show little relationship with reaction coordinate. At pH 6.0, the 82% and 83% protonation ratio would result in the four TLR3 molecules gaining a total of 75.1 and 76.4 protons (18.8 and 19.1 protons for each TLR3), leading to the TLR3 molecules in the signaling complex exhibiting an overall positive charge of ≈+10.8 and +11.1 (the un‐titrated residues in TLR3 exhibits ‐8 negative charge).

Meanwhile, only 49% and 48% of the titrated residues were protonated at pH 7.4. Thus, those titrated residues in tetramer only possess 45.1 and 44.2, which indicates the TLR3 monomer molecules in the signaling complex only exhibiting an overall positive charge of ≈+3.3 and +3.0. That is, the raising of system pH from 6.0 to pH 7.4 might decrease ≈ 70% on the total charge. Since the positive charge on TLR3 is fundamental for its recognition of negatively charged dsRNA, the decreased positive of TLR3 would decrease the stability of the signaling complex.

#### 3.3.2 Fingerprint of Charged Histidines

**Figure 6:**
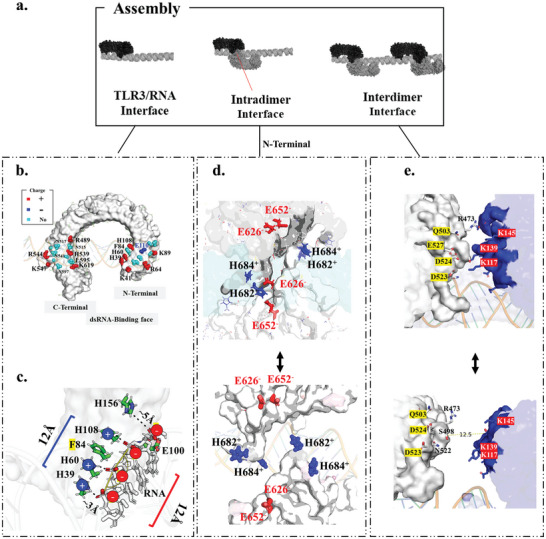
Charged fingerprints relevant to the assembly of signaling complexes. a) Possible assembly process for TLR3 on dsRNA and the resulting interface. b) Residues positioned on the TLR3/dsRNA interface, color‐coded according to their potential charge states. c) Zoom‐in view of the fingerprint at the N‐terminal of TLR3 concerning RNA binding. d) Zoom‐in view of the fingerprint at the C‐terminal of TLR3 for dimerization. e) Zoom‐in view of the fingerprint for multimerization.

The protonation ratio of histidine residues in different locations of TLR3 is uneven (as shown in Figure 5 and detailed in Table 1). Some histidine residues at the assembling interface show high protonation ratio (≈100%) even in pH 7.4 condition, which indicates certain “fingerprint” formed by histidine for assembling. That mainly involves, three histidine residues at the N‐terminal (H39, H60, and H108) and one H539 residue nears the C‐terminal of TLR3 (Figure 5e–h) for dsRNA binding, and the H682 and H684 at the C‐Terminal of TLR3 for dimerization. This high level of protonation effectively keeps these histidine residues positively charged, thereby promoting the formation of ionic bonds with the negatively charged molecular such as dsRNA. Moreover, the entire TLR3 face for dsRNA binding is rich of positive charged residues but contains only one negatively charged residue (E110, **Figure** **6**a,b). All the fingerprints for assembling were shown in Figure 6b–d.

#### 3.3.3 Cation‐π Interaction in Fingerprint at TLR3/dsRNA Interface

The TLR3/dsRNA interface is rich in histidine residue to form ionic bond with dsRNA. Intriguingly, the histidine/R_2_PO─O^─^ interaction fingerprint at the N‐terminal (Figure 6c) contain three phosphate groups (R_2_PO─O^─^/R_2_PO─O^─^/ R_2_PO─O^─^) and three histidine residues, but one more residue (Phe84) is inset into the three histidine residues to form His39^+^/His60^+^/Phe84/His108^+^ group. Both of those interaction groups on TLR3 and dsRNA are ≈12Å in length scale, facilitating a perfect fit for interaction for each other. The three histidine residues manage to establish ionic bonds phosphate groups of dsRNA with the histidine residues, facilitated by the bonds' rotational flexibility.

Yet, the inclusion of the uncharged Phe84 in this dsRNA‐binding group at the N‐terminal of TLR3 raises a significant question. Phe84 may primarily contribute through its ability to engage in aromatic‐aromatic (π‐π) stacking interactions to join and extend the His39^+^/His60^+^/His108^+^ group to ≈12 Å, which is required for fitting the scale of three R_2_PO‐O^−^ in ligand dsRNA. The inset of Phe84 between histidine could also reduce electrostatic repulsion thus preventing those histidine residues from being unprotonated.

**Figure 7:**
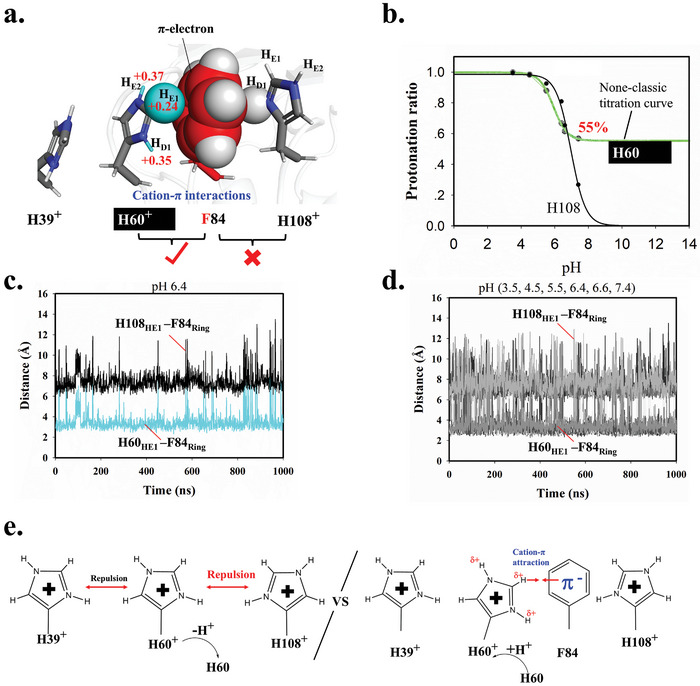
The cation‐π interactions at the N‐terminal region of TLR3. a) Different levels of protonation ability for H60 and H108 in free TLR3, as described by the titration curves from the CpHMD simulation. b) A cation‐π interaction is observed to occur between the H_E1_, H_E2_, and H_D1_ atoms of histidine residue H60^+^ and the aromatic ring atom of F84, with lesser involvement noted for the cation‐π interaction between H108^+^ and F84. The H_E1_ atom in H60 and the ring atom in F84 are depicted in spheres for better visualization. Charge distribution among the atoms in [H39‐H60‐F84‐H108]^3+^ cluster was calculated using the # M062x/6‐31G** sp level by Gaussian09.^[^ ^62^ ^]^ The coordinates were extracted from a snapshot representing the S_0_ state, identical to the structure in Figure 4f. c) Distance between the H_E1_ atom of H60/H108 and the ring carbon of F84 in free TLR3 at pH 6.4. d) Distance between the H_E1_ atom of H60/H108 and the ring carbon of F84 in free TLR3 at pH 3.5, 4.5, 5.5, 6.4, 6.6, and 7.4. e) Proposed mechanism for F84 residues to stabilize the three nearby protonated histidine clusters with an obvious positive charge by cation‐π interaction. The statistical analysis for atom distance was performed using CPPTRAJ module from AmberTools.

Another role of Phe84 in His39^+^/His60^+^/Phe84/His108^+^ cluster is to engage cation‐π interaction^[^ ^63^ ^]^ with charged histidine, thereby promoting the ratio of charged histidine and facilitating dsRNA binding. Cation‐π interaction involves Coulomb attractive forces between positively charged atom groups and the π‐electron cloud of aromatic groups.^[^ ^63^ ^]^ As shown in Figure S5 (Supporting Information), all the histidine ring hydrogen atoms exhibit positive charge. The positive charged H_E1_, H_E2_, and H_D1_ atoms in His60^+^ can successfully form cation‐π interaction with the aromatic ring of Phe84. In this arrangement, the H_E1_ atom is positioned at the center of the aromatic ring of Phe84, while the H_E2_ and H_D1_ atoms also engage with the aromatic ring of Phe84 (**Figure** **7**a). This strong Coulomb attractive force stabilizes charged His60^+^, thereby promoting the protonation ratio of His60. In other words, the transformation of His60^+^ into uncharged His60 must overcome this cation‐π interaction, making it unfavorable within TLR3. Consequently, the cation‐π interaction between His60^+^ and Phe84 causes the His60 residues to exhibit a 55% protonation ratio in a basic environment (Figure 7b). However, as depicted in Figure 7c, the histidine His108 on the opposite side of Phe84 fails to form a cation‐π interaction with Phe84, because the geometry only allows one H_D1_ atom to interact with the aromatic ring of Phe84, resulting in a standard titration curve for His108 (Figure 7b; Figure S2, Supporting Information).

#### 3.3.4 Dipole–Dipole Interaction and Fingerprint at Intradimer Interface

As shown in Figure 6c, the C‐terminal of the TLR3 tail, which is responsible for the dimerization of TLR3, is also rich in histidine, including H674, H882, and H684. Although the protonation ratio of these C‐terminal histidine residues is lower than those at the N‐terminal interacting with dsRNA (nearly 100%), they are still significantly protonated at pH 6.0 with higher‐than‐average value of 82%/83% (Figure 5e,g). This suggests that these histidine residues predominantly maintain a +1 positive charge state. Moreover, charged histidine residues and Coulomb interactions are pivotal in dimerization processes. As illustrated in Figure 6d, the C‐terminal of TLR3 features a dipole formed by the positively charged histidine H682^+^/H684^+^ and negatively charged E652^−^ /E626^−^, facilitating dipole‐dipole interactions during the antisymmetric assembly of two TLR3 molecules.

Moreover, the histidine residues at the intradimer TLR3/TLR3 interface tend to be unprotonated (<40%) when pH raised to 7.4 (Figure 5f,h), which disrupting the dipole‐dipole interaction that depends on the H682^+^/H684^+^—E626^−^/E652^−^ dipole. This is the reason why dimerization of TLR3 requires acid environment and is the reason why different system pH result in different PMF landscape in Figure 4.

## 4 Conclusion

The pH‐dependent assembly of proteins to produce a threshold‐like response is a fundamental principle in cell signaling. Using constant protonation states during simulations based on a single set of p*K*_a_ values predicted by software such as PROPKA can be problematic. This is because even if we disregard errors in p*K*_a_ predictions, some residues may experience significant p*K*_a_ shifts during reactions. The formation or disruption of strong interactions involving a specific protonation state of a titratable residue can lead to substantial p*K*_a_ shifts for that residue. Assigning fixed protonation states to all titratable residues using the CpHMD method can be computationally expensive due to the scale of the biosystem and the number of titratable residues. Therefore, we implemented a hybrid CpHMD/traditional simulation approach under the instruction of two sets of p*K*_a_ values: one for the initial state and one for the final state. A residue is assigned a constant protonation state only if both sets of p*K*_a_ values agree on its protonation state.

In this study, we investigated the pH‐dependent stability of the TLR3/dsRNA signaling complex using a hybrid CpHMD/traditional simulation approach. Our simulations replicated experimental findings, demonstrating that the TLR3 signaling complex remains stable only under acidic conditions while becoming unstable in basic environments. Further analysis revealed that the stability of the signaling complex is primarily governed by charged histidine residues. The Coulombic interactions between positively charged histidines and negatively charged dsRNA are crucial. Additionally, specific charged “fingerprints” play a significant role in driving the complex assembly into a highly ordered structure. Histidine residues at the C‐terminal form a pH‐dependent dipole [H682^+^/H684^+^]‐[E626^−^/E652^−^], facilitating the antisymmetric dimer formation through dipole‐dipole interactions. At the N‐terminal, three charged histidine residues [His39^+^, His60^+^, His108^+^] and an uncharged aromatic residue, Phe84, form a pH‐dependent positively charged cluster. Phe84 helps align three phosphate groups in dsRNA for optimal ligand binding and engages in cation‐π interactions with His60^+^.

These findings provide insight into the general pH‐dependent phenomena. The hybrid CpHMD/traditional simulation approach may be applicable to other systems as well. The formation of specific charged “fingerprints” could be a universal feature in highly ordered macromolecular assemblies. The implications of our work extend beyond TLR3, providing a general framework for understanding how pH can influence the protonation and charge states of titratable residues, forming specific interaction patterns critical for protein assembly and function.

## Conflict of Interest

The authors declare no conflict of interest.

## Author Contributions

P.L. conceived and designed the experiments, performed the experiments, analyzed the data, and wrote the original manuscript. M.S., Y.W., Q.L., and X.D. helped with the experiment design and revised the manuscript. X.W. conceived and designed the experiments, supervised the study, and revised the manuscript.

## Supporting information



Supporting Information

Supplemental Video 1

## Data Availability

The data that support the findings of this study are available in the supplementary material of this article.
