# Supplementary material for: pH‐Dependent Assembly and Stability of Toll‐Like Receptor 3/dsRNA Signaling Complex: Insights from Constant pH Molecular Dynamics and Metadynamics Simulations
Source: Adv Sci (Weinh). 2024 Nov 8;12(1):2411445. doi: 10.1002/advs.202411445 (PMC11714240; doi:10.1002/advs.202411445)
Supplement: Supplementary file 1 — Supporting Information [file ADVS-12-2411445-s001.pdf]

ADVANCED SCIENCE
Open Access

Supporting Information

for *Adv. Sci.*, DOI 10.1002/advs.202411445

pH-Dependent Assembly and Stability of Toll-Like Receptor 3/dsRNA Signaling Complex: Insights from Constant pH Molecular Dynamics and Metadynamics Simulations

Penghui Li, Mingsong Shi, Yibo Wang, Qiong Liu, Xiubo Du and Xiaohui Wang**

Support Information for:

pH-Dependent Assembly and Stability of Toll-Like Receptor 3/dsRNA Signaling Complex: Insights from Constant pH Molecular Dynamics and Metadynamics Simulations

Penghui Li[1,2], Mingsong Shi[3], Yibo Wang[4], Qiong Liu[1,2,5], Xiubo Du[1]*, Xiaohui Wang[4,6]*

[1]Shenzhen Key Laboratory of Marine Biotechnology and Ecology, College of Life Sciences & Oceanography, Shenzhen University, Shenzhen 518055, China
[2]Key Laboratory of Optoelectronic Devices and System of Ministry of Education and Guangdong Province, College Physics and Optoelectronic Engineering, Shenzhen University, Shenzhen 518060, China
[3]NHC Key Laboratory of Nuclear Technology Medical Transformation, Mianyang Central Hospital, School of Medicine, University of Electronic Science and Technology of China, Mianyang, Sichuan, 621099, China
[4]Laboratory of Chemical Biology, Changchun Institute of Applied Chemistry, Chinese Academy of Sciences, Changchun, Jilin, 130022, China
[5]Shenzhen-Hong Kong Institute of Brain Science, Shenzhen Fundamental Research Institutions, Shenzhen 518055, China
[6]School of Applied Chemistry and Engineering, University of Science and Technology of China, Hefei, 230026, China

* Corresponding authors
Emial: duxiubo@szu.edu.cn；xiaohui.wang@ciac.ac.cn

Contents

Figure S1. Convergence analyses of the four metadynamics simulations, which were characterized by block analysis (follow the instruction at https://www.plumed.org/doc-v2.9/user-doc/html/master-_i_s_d_d-2.html) and the PMF evolution along the simulation time. The units for energy are in kJ/mol. 3

Figure S2. Simulated titration curves of residues in free TLR3 in water. Estimated pK_a value were labeled (location with 50% protonation ratio in curve). 4

(Continued) Figure S2. Simulated titration curves of residues in free TLR3............. 5

(Continued) Figure S2. simulated titration curves of residues in free TLR3. 6

(Continued) Figure S2. Simulated titration curves of residues in free TLR3............. 7

(Continued) Figure S2. Simulated titration curves of residues in free TLR3............. 8

Figure S3. Location of histidine residues in TLR3 protein. The histidines located around assembling interface are colored in red, while the other histidines are colored in black. 9

Figure S4. One-dimensional minimum free energy path (MEP, middle) for the free energy landscape in Figure 4g (left) and Figure 4j(right) along RC3......................... 10

Figure S5. Charge distribution among the atoms in $[H39\text{-}H60\text{-}F84\text{-}H108]^{3+}$ cluster was calculated using the M062x/6-31G(d,p) sp level by Gaussian09. The coordinates were extracted from a snapshot representing the S0 state, identical to the structure in Figure 4f. 11

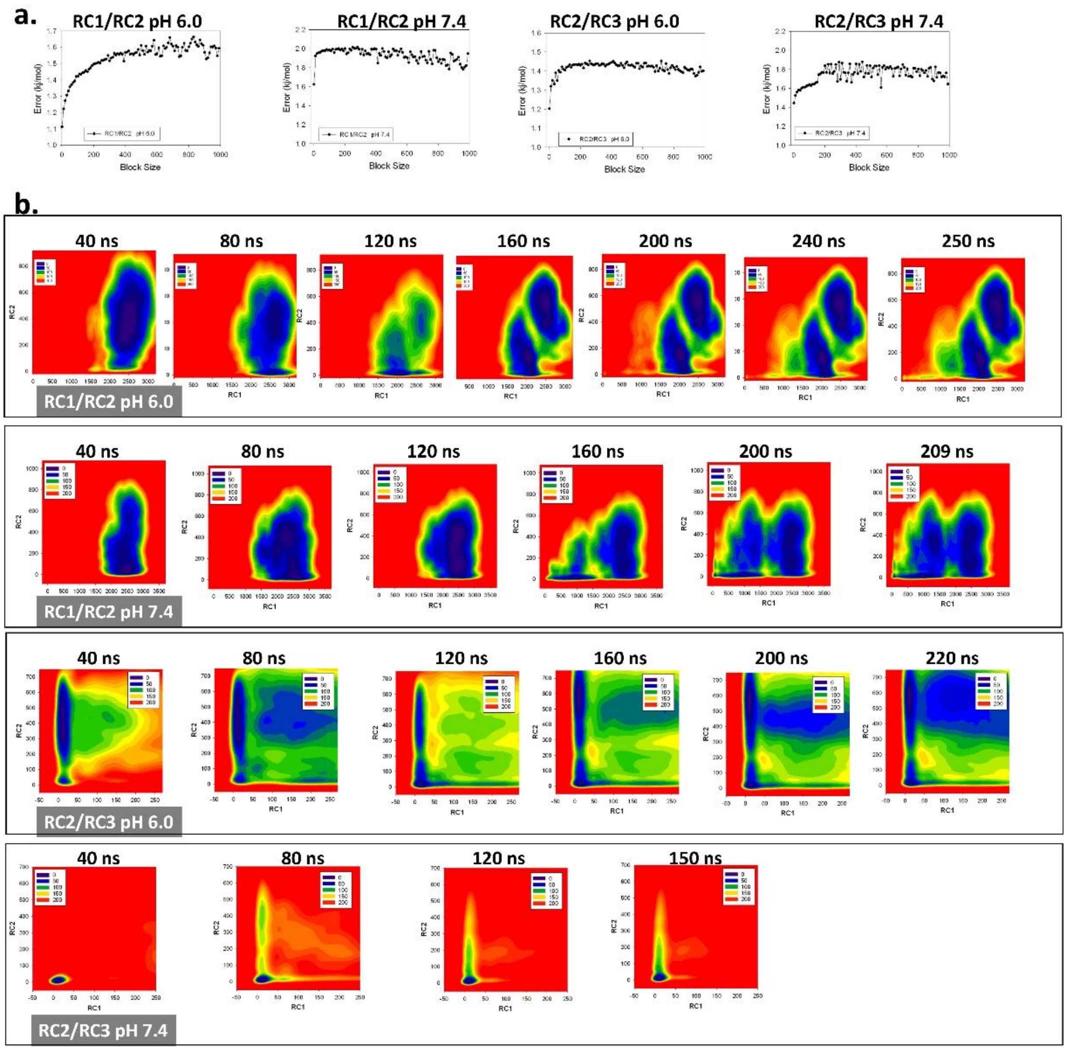

Figure S1. Convergence analyses of the four metadynamics simulations, which were characterized by block analysis (follow the instruction at https://www.plumed.org/doc-v2.9/user-doc/html/master-_i_s_d_d-2.html) and the PMF evolution along the simulation time. The units for energy are in kJ/mol.

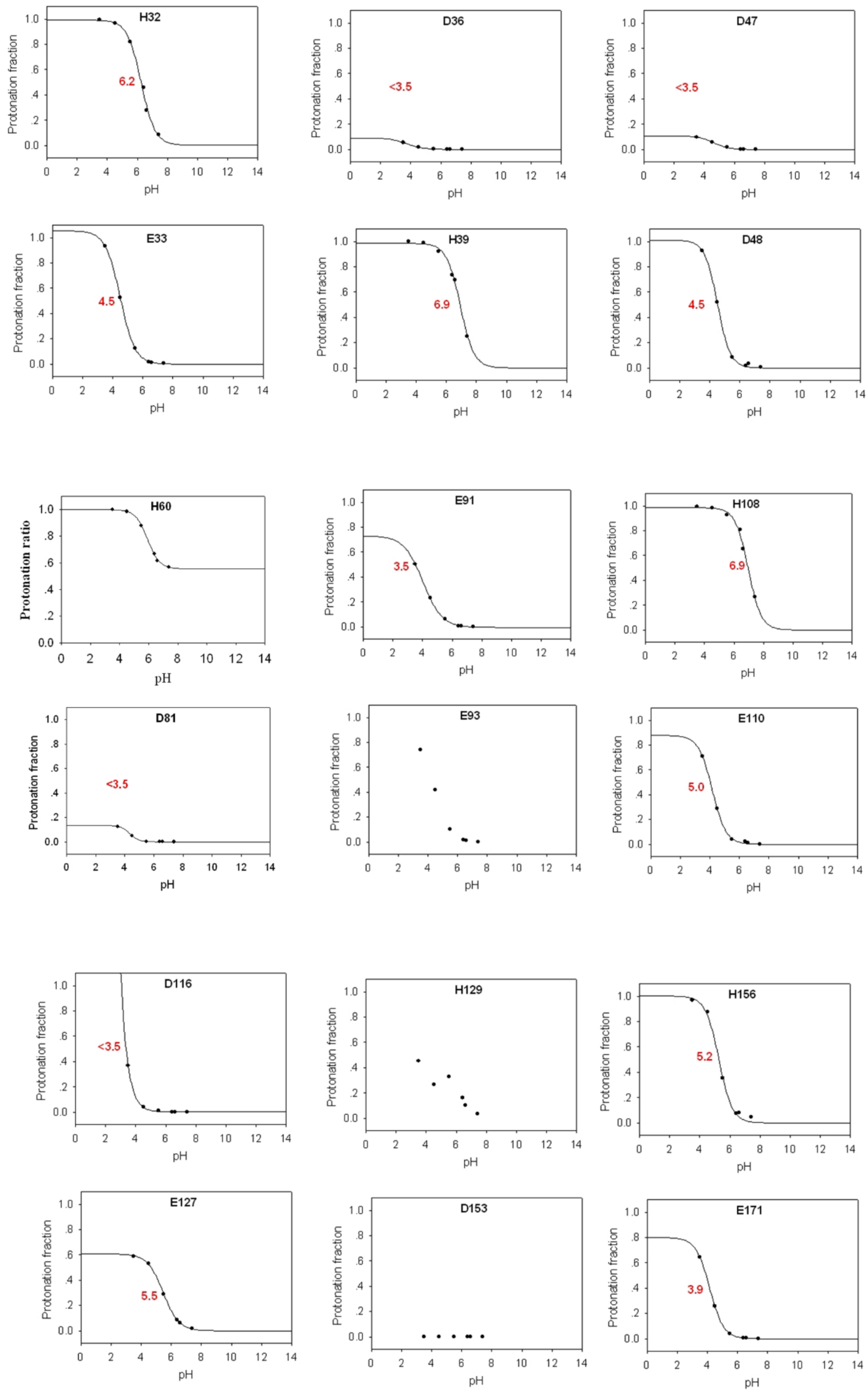

Figure S2. Simulated titration curves of residues in free TLR3 in water. Estimated pK_a value were labeled (location with 50% protonation ratio in curve).

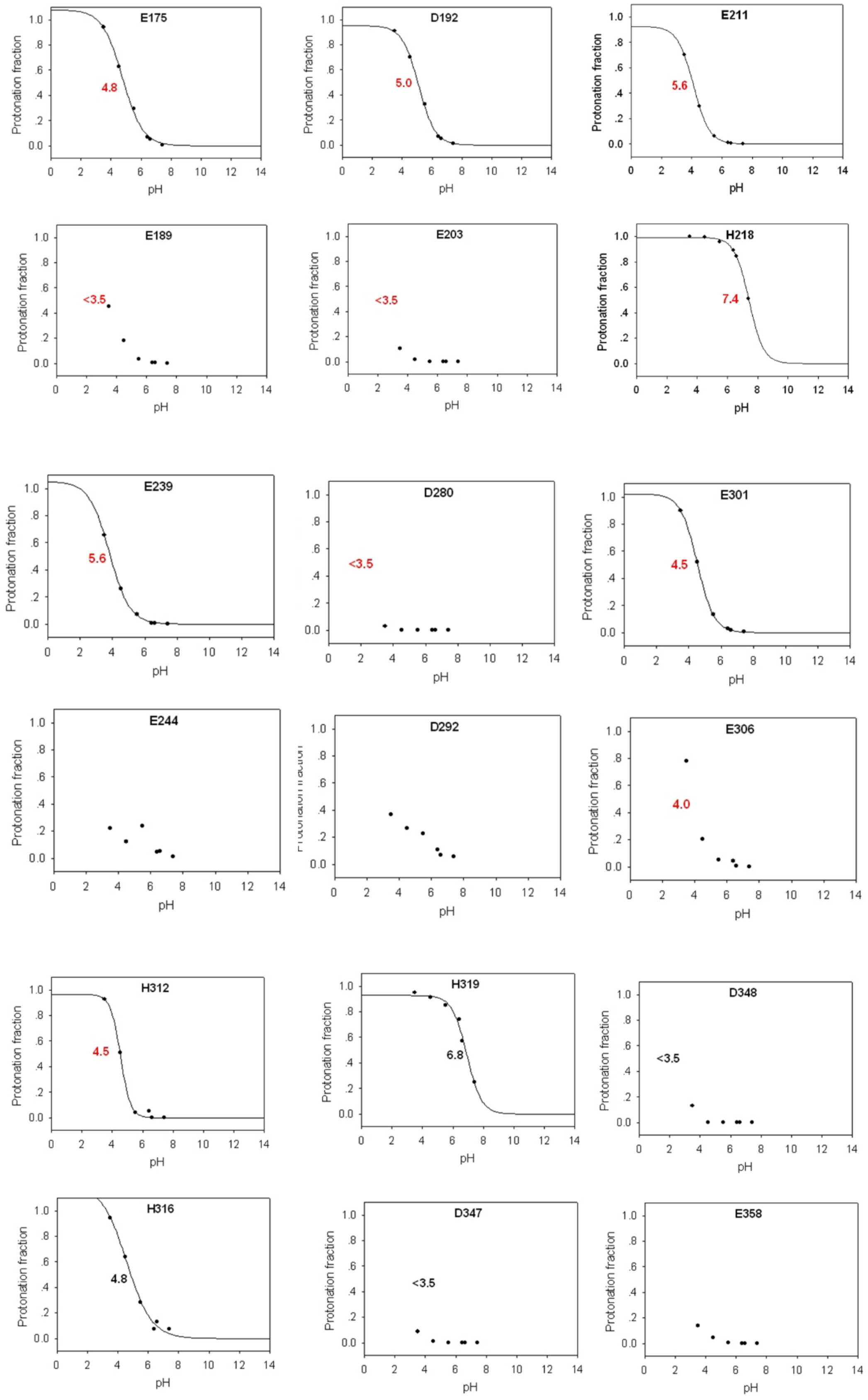

(Continued) Figure S2. Simulated titration curves of residues in free TLR3.

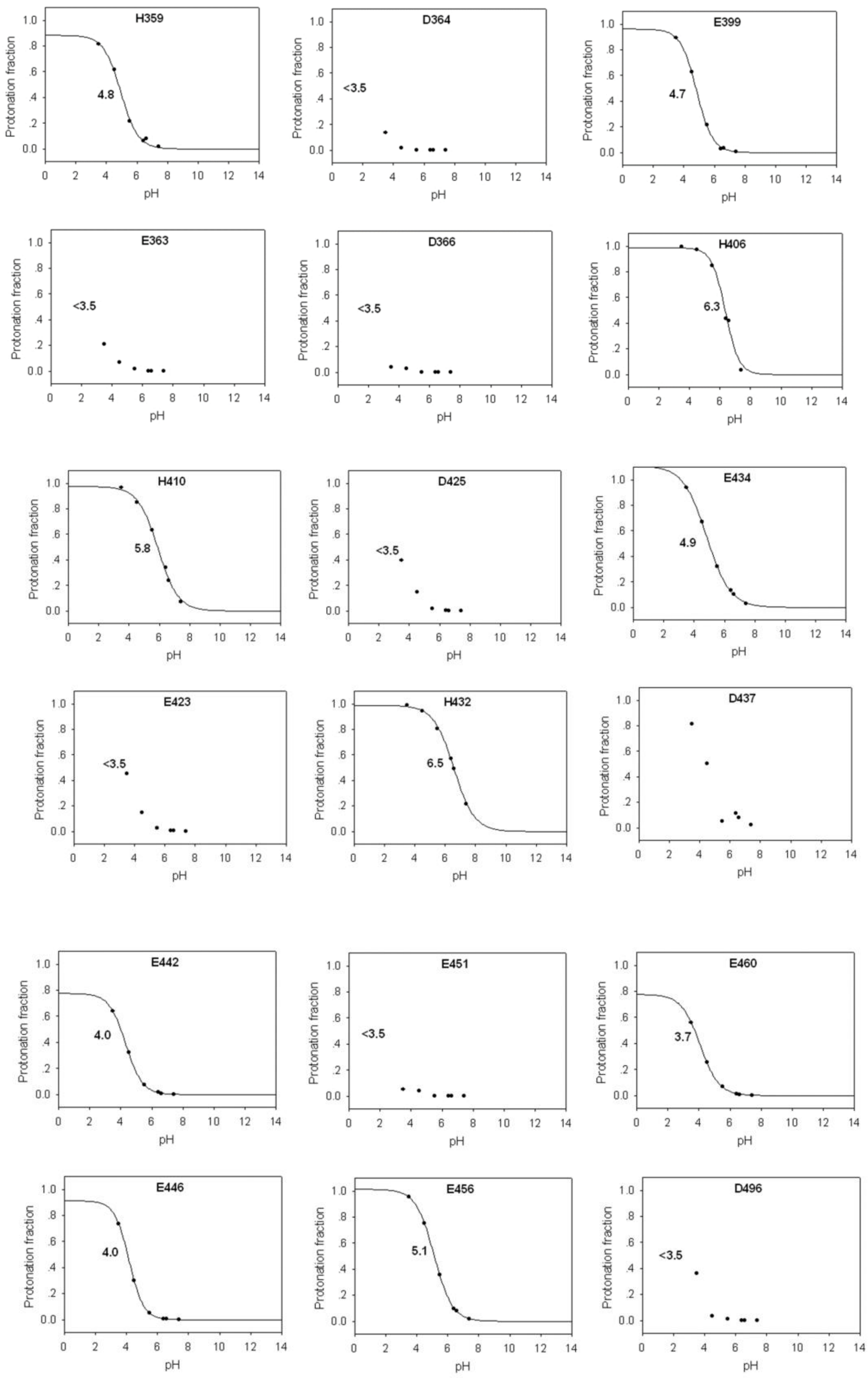

(Continued) Figure S2. simulated titration curves of residues in free TLR3.

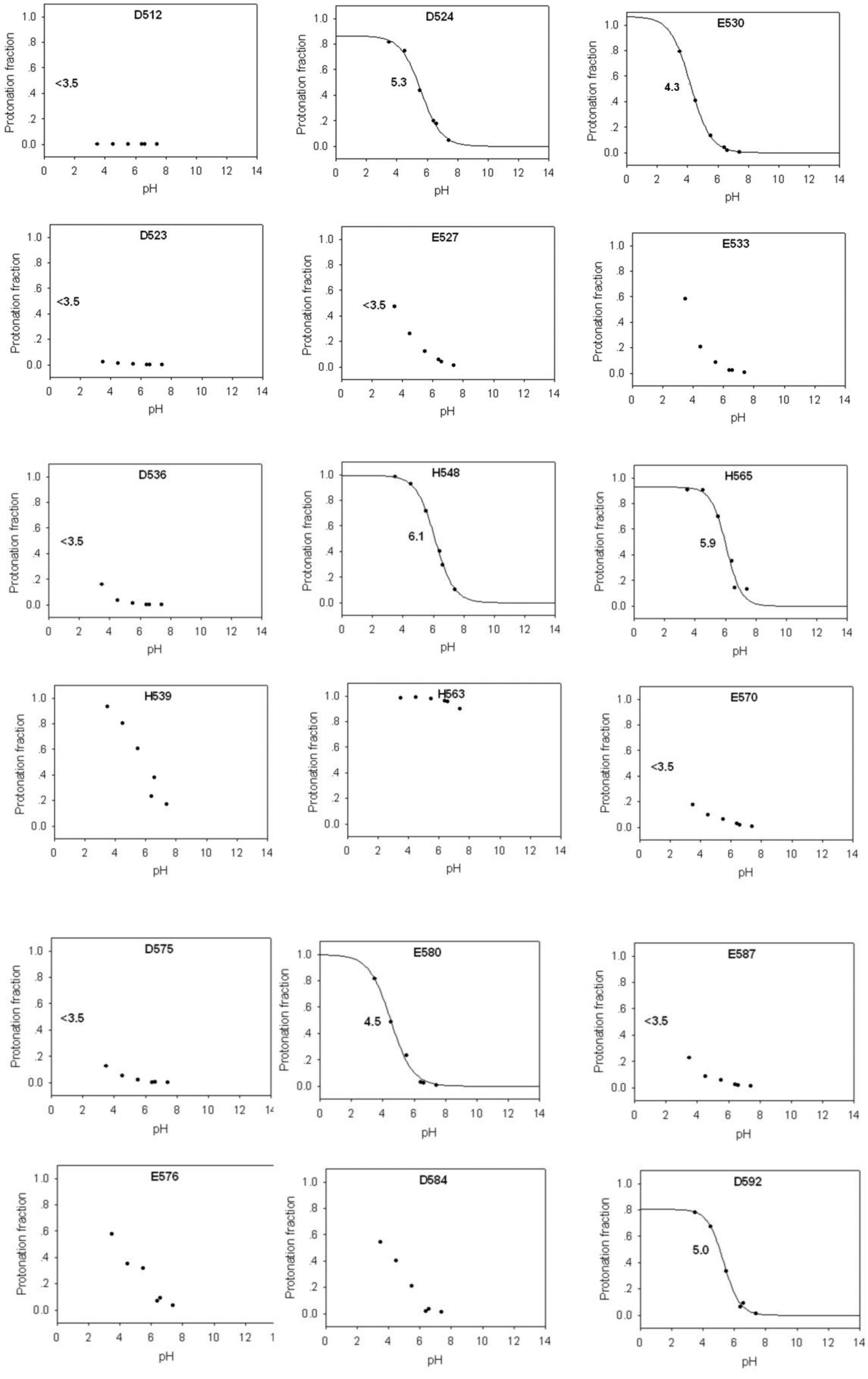

(Continued) Figure S2. Simulated titration curves of residues in free TLR3.

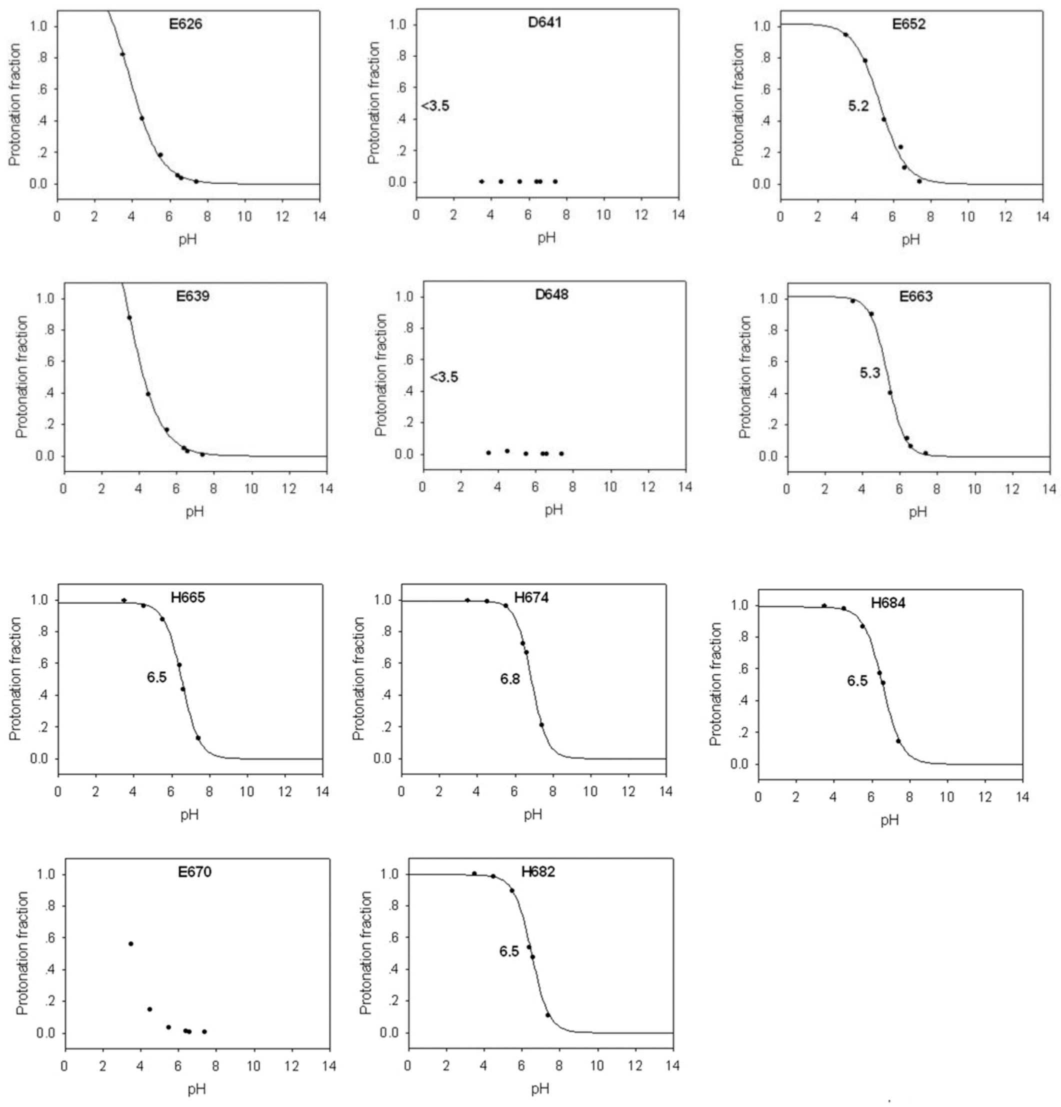

(Continued) Figure S2. Simulated titration curves of residues in free TLR3.

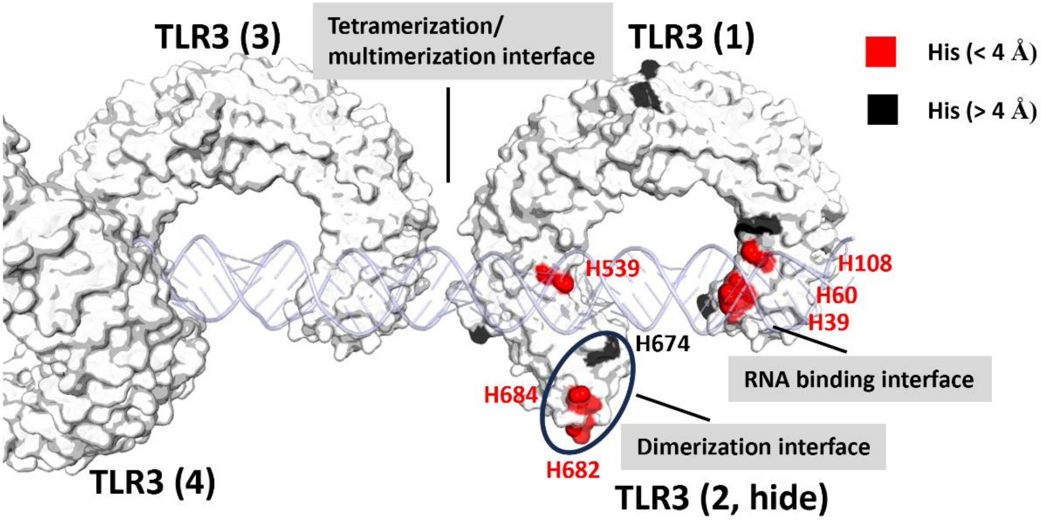

Figure S3. Location of histidine residues in TLR3 protein. The histidines located around assembling interface are colored in red, while the other histidines are colored in black.

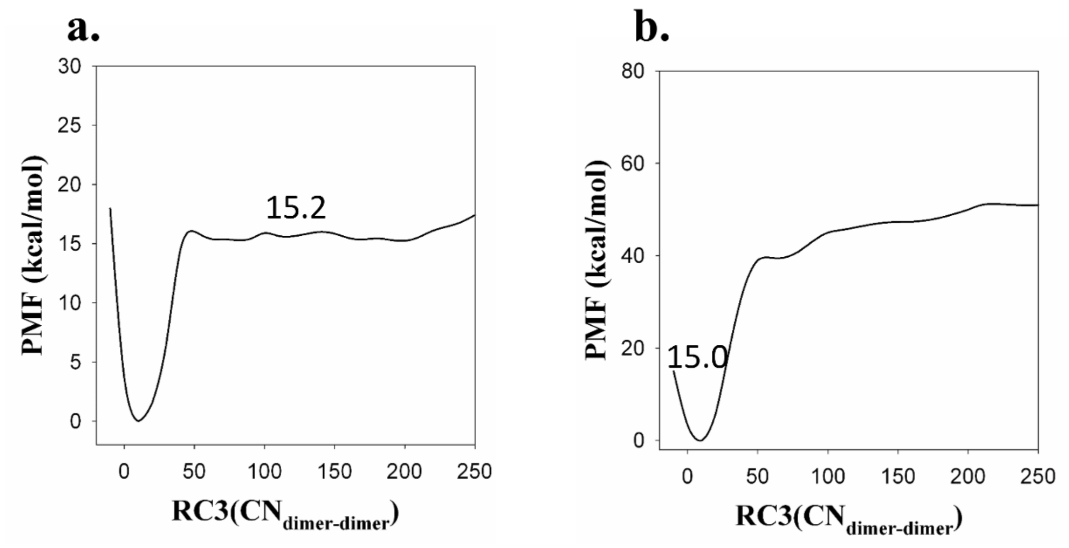

Figure S4. One-dimensional minimum free energy path (MEP, middle) for the free energy landscape in Figure 4g (left) and Figure 4j(right) along RC3.

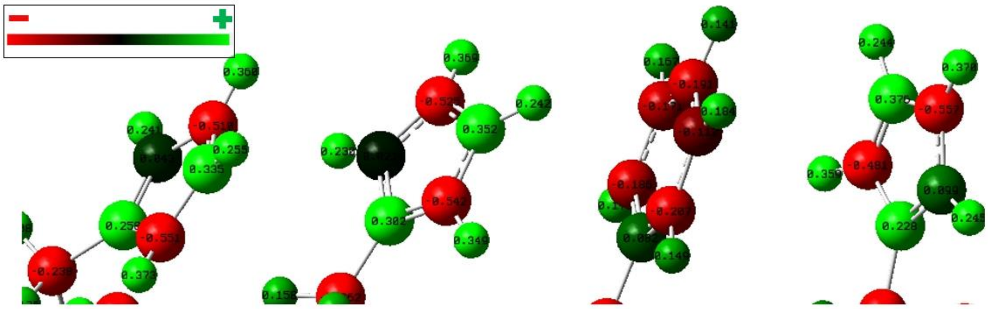

H39$^+$ **H60$^+$** **F84** **H108$^+$**

Figure S5. Charge distribution among the atoms in [H39-H60-F84-H108]$^{3+}$ cluster was calculated using the M062x/6-31G(d,p) sp level by Gaussian09. The coordinates were extracted from a snapshot representing the S0 state, identical to the structure in Figure 4f.
